# ^18^F-FDG PET/CT metabolic parameters can semi-quantitatively evaluate the nature of the heart and pericardial masses: a retrospective study

**DOI:** 10.1038/s41598-024-67336-8

**Published:** 2024-07-15

**Authors:** Xianwen Hu, Peiqing Yang, Dongfeng Pan, Pan Wang

**Affiliations:** Department of Nuclear Medicine, Affiliated Hospital of Zunyi Medical University, 149 Dalian Road, Huichuan District, Zunyi, 563000 China

**Keywords:** PET/CT, Metabolic parameters, Cardiac tumor, Pericardial mass, Diagnostic performance, Cancer, Cancer imaging

## Abstract

The objective of this study was to evaluate semi-quantitatively the diagnostic performance of PET/CT metabolic parameters in differentiating benign or malignant cardiac or pericardial masses. A total of forty-one patients with newly diagnosed cardiac/pericardial masses who underwent ^18^F-FDG PET/CT were recruited. PET/CT metabolic parameters including the maximum standardized uptake value (SUVmax), mean standardized uptake value (SUVmean), total lesion glycolysis (TLG), tumor metabolic volume (MTV), the maximum tumor-to-mediastinal background ratio (TMR) and the maximum tumor-to-liver background ratio (TLR) is measured or calculated to evaluate the benign or malignant nature of cardiac/pericardial masses. Compared with benign cardiac/pericardial lesions, cardiac/pericardial malignancies had higher SUVmax, SUVmean, TLG, MTV, TMR, and TLR. All these PET/CT metabolic parameters showed high diagnostic performance in semi-quantitative evaluation of benign or malignant cardiac or pericardial masses, and SUVmean and MTV had the highest diagnostic accuracy. Therefore, PET/CT metabolic parameters can semi-quantitatively evaluate the benign or malignant cardiac/pericardial masses.

## Introduction

Clinically, cardiac tumors are rare, and the incidence of primary cardiac tumors is only 0.0017–0.02%^1^. For primary cardiac tumors, 75% are benign, such as myxoma, hemangioma, etc., and about 25% of cardiac tumors are malignant, mostly angiosarcoma and rhabdomyosarcoma^2^. Compared with the primary cardiac tumors, secondary cardiac tumors are more common, and most of them are malignant tumors, such as metastatic tumors, lymphomas^3^. The treatment methods for benign and malignant cardiac tumors are different, and there are significant differences in prognosis, so it is essential to obtain an accurate diagnosis before treatment for patients to obtain the best treatment plan. For primary benign cardiac tumors or malignant cardiac tumors with limited lesions and no distant metastasis, surgical resection is the main treatment method. However, as for secondary cardiac malignancies, surgical treatment is often not possible and chemotherapy/radiotherapy is usually treated^4^.

Traditional imaging methods, including echocardiography, CT and MRI, can evaluate the morphological characteristics of cardiac tumors and provide certain diagnostic value for the differentiation of benign and malignant cardiac tumors^5–7^. ^18^F-FDG PET/CT, which integrates morphological and metabolic function information, has been widely used in the diagnosis, preoperative staging, treatment and prognosis assessment of tumors, and has shown higher clinical application value than traditional imaging methods in the management of tumors^8,9^. The maximum standardized uptake value (SUVmax), mean standardized uptake value (SUVmean), total lesion glycolysis (TLG), tumor metabolic volume (MTV), the maximum tumor-to-mediastinum (blood pool of descending aorta) background ratio (TMR) and the maximum tumor-to-liver background ratio (TLR) as important metabolic parameters in PET/CT, have shown high diagnostic value in the differentiation of benign and malignant tumors^10–12^. Due to the rarity of cardiac tumors, there is currently limited research on the diagnosis and differentiation of cardiac tumors through PET/CT^13–15^. This study retrospectively analyzed the ^18^F-FDG PET/CT imaging data of 35 patients with cardiac tumors, aiming to explore the diagnostic value of ^18^F-FDG PET/CT metabolic parameters in the diagnostic value of cardiac tumors.

## Material and methods

This retrospective study received approval from the institutional review board of the Affiliated Hospital of Zunyi Medical University, and all methods were performed in accordance with the relevant guidelines and regulations.

### Patients

Patients with suspected cardiac or pericardial lesions who received ^18^F-FDG PET/CT from December 2019 to Ocot 2023 were retrospectively analyzed. Inclusion criteria: (i) PET/CT images of patients with suspected heart or pericardial lesions are clear and do not affect interpretation; (ii) Patients had no history of prior tumor-related surgeries or other treatments before examination; (iii) The final lesions were confirmed by surgery or puncture pathology, and the lesions that could not obtain pathology were confirmed by other imaging follow-up, and the follow-up time was at least more than 2 years; and (iv) the clinical data of the patients were complete.

### ^18^F-FDG PET/CT imaging

The production and synthesis of ^18^F-FDG utilized the HM-10HP cyclotron (Sumitomo Corporation, Japan) and F300E modular automatic synthesis device of the FDG chemical synthesis system. Imaging commenced after intravenous injection when the radiochemical purity of ^18^F-FDG exceeded 95%. Intravenous administration of ^18^F-FDG (0.1–0.15 mCi/kg) occurred after a 6-h fast, and when the patient’s blood glucose level was < 11.1 mmol/L. Imaging took place 50 to 65 min post-injection using the Biograph mCT PET/CT scanner (Siemens, Germany). The scanning range varied from the top of the skull to the middle of the femur or vertex to the feet based on the patient’s diagnosis and clinical presentation. CT scanning employed a tube voltage of 120 kV, tube current of 119 mA, and a slice thickness of 5 mm. PET scanning was conducted immediately after CT completion, with a scanning parameter of 2 min per bed over 6 to 7 beds. PET images underwent attenuation correction with CT data and were reconstructed using the TrueX + TOF method post-image acquisition.

### Imaging analysis

The medical image processing software of Shanghai Xingxiang Medical Device Co., Ltd. (Shanghai, China) was used for image analysis. In ^18^F-FDG PET/CT images, select the layer with the largest ^18^F-FDG uptake area in tumor tissue for delineation; then manually draw an elliptical region of interest (ROI) at this level, to ensure that it has sufficient edges and completely surrounds the entire tumor. Once the boundary is determined, the software automatically outlines the volume of interest (VOI) of the tumor tissue. After defining the boundaries, the software automatically calculates the VOI’s SUVmax and SUVmean. MTV and TLG are calculated using a fixed percentage SUVmax threshold method. VOI is defined as the area higher than 40% SUVmax, where MTV is the volume of VOI, and TLG is the product of MTV and SUVmean. For the density of the lesion on CT, low-density cystic necrosis and high-density hemorrhage and calcification in nodules or masses of the heart or pericardium are defined as heterogeneous, while homogeneous density is considered if none of the above occurs.

All PET/CT images were interpreted independently by two attending nuclear medicine physicians, each possessing over 5 years of experience in PET/CT imaging diagnosis. In the event of discrepancies or disagreements, both physicians engaged in negotiations until a consensus was achieved. Semi-quantitative analysis involved calculating the SUVmax, SUVmean, TLG, MTV, TMR and TLR for the VOI in patients with cardiac lesions.

### Statistical analysis

The statistical analysis was performed using SPSS version 29.0 (IBM Corporation, Armonk, NY). The Shapiro–Wilk test is used to evaluate the normality of continuous variables. For normally distributed data, expressed as mean ± standard deviation. For non-normally distributed data, the statistical description was presented as the median (Q1, Q3), and between-group comparisons were performed using the independent samples rank sum test. Categorical data were presented as the number of cases (%) for count variables, and between-group comparisons were carried out using the chi-square test. In cases where the conditions for the chi-square test were not met, Fisher’s exact probability method was applied. All tests were two-sided, and statistical significance was considered when *p* < 0.05.

### Ethics approval and consent to participate

This retrospective study received approval from the institutional review board of Zunyi Medical University’s afliated hospital, and all methods were performed in accordance with the relevant guidelines and regulations. Written informed consent was obtained from all subjects and/or their legal guardian(s).

## Results

### Clinical and ^18^F-FDG PET/CT features of patients with cardiac and pericardial lesions

A total of forty-one patients, including 22 males and 19 females, were retrospectively analyzed according to the inclusion criteria. Based on the pathologic nature of the tumor, the patients were divided into benign (n = 18) and malignant group (n = 23), and the baseline characteristics and PET/CT findings of the patients are summarized in Table 1.

**Table 1:** Clinical and PET/CT features of patients with cardiac or pericardial masses.

Parameters	Benign group	Malignant group	*p* value
Age (years)	46 ± 18	52 ± 17	0.271
Gender			
Man	8 (44.4%)	14 (60.9%)	0.639
Woman	10 (55.6%)	9 (39.1%)	
Density			
Homogeneous	11 (61.1%)	9 (39.1%)	0.129
Heterogeneous	7 (38.9%)	14 (60.9%)	
Hydropericardium			
Yes	7 (38.9%)	12 (52.2%)	0.542
Not	11 (61.1%)	11 (47.8%)	
SUVmax	3.35 ± 2.46	13.31 ± 8.99	** < 0.001**
SUVmean	2.03 ± 1.51	8.50 ± 5.13	** < 0.001**
MTV (cm^3^)	10.37 ± 6.57	36.99 ± 16.00	** < 0.001**
TLG (g)	11.23 [6.65, 36.52]	242.43 [167.64, 393.06]	** < 0.001**
TMR	1.81 ± 1.63	6.72 ± 4.88	**0.0002**
TLR	1.26 ± 1.07	5.54 ± 4.65	**0.0005**

SUVmax, maximum standard uptake value; SUVmean, mean standard uptake value; MTV, metabolic tumor volume; TLG, total lesion glycolysis; TMR, the maximum tumor-to-mediastinal background ratio; TLR, the maximum tumor-to-liver background ratio. Significant values are in bold.

Of the 18 benign cases, the most common was myxoma (5 cases), followed by hemangioma (3 cases), tumorous lesions, including nonspecific inflammation (2 cases, diagnosed with pericarditis were confirmed by ultrasound or/and CT examination after anti-inflammatory therapy and the lesion disappeared), tuberculous pericarditis (2 cases), thrombus (1 case), ventricular aneurysm (1 case) and angioleiomyolipoma (1 case). The remaining 3 patients with benign tumors did not have a clear histopathological type and were identified by follow-up, which showed no significant changes in the lesions during a follow-up period of at least 28 months. In the malignant group, the most common pathological type was angiosarcoma (8 cases), followed by diffuse large B-cell tumors (5 cases), peripheral T cell lymphoma (1 case), metastases (3 cases), mesothelioma (2 cases), perihemangiocytoma (1 case), myxomatodes sarcoma (1 case), rhabdomyosarcoma (1 case) and fibrosarcoma (1 case). The histologic distributions and anatomic locations of these patients are summarized in Table 2.

**Table 2:** Histologic types and lesion locations of the enrolled 41 patients with cardiac/pericardial masses.

Histology type	No. of patients (%)	Location	Method of diagnosis
Malignant	23 (56.1%)		
Angiosarcoma	8 (19.5%)	Left atrium (2), right atrium (5), pericardium (1)	Surgery (7),
			Biopsy (1)
Diffuse large B-cell lymphoma	5 (12.2%)	Left atrium (2), right atrium (1), right atrium + postcava (1), pericardium (1)	Surgery (3)
			Biopsy (2)
Peripheral T cell lymphoma	1 (2.4%)	Pericardium + right atrium	Biopsy
Mesothelioma	2 (4.9%)	Pericardium	Biopsy
Perihemangiocytoma	1 (2.4%)	Left ventricle	Surgery
Myxomatodes sarcoma	1 (2.4%)	Left atrium	Surgery
Rhabdomyosarcoma	1 (2.4%)	Left ventricle	Surgery
Fibrosarcoma	1 (2.4%)	Left ventricle	Surgery
Lung cancer metastasis	2 (4.9%)	Right atrium + pericardium (1), right atrium + right pulmonary vein (1)	Biopsy
Thyroid cancer metastasis	1 (2.4%)	Pericardium	Biopsy
Benign	18 (43.9%)		
Myxoma	5 (12.2%)	Left atrium (2), right atrium (2), left ventricle (1)	Surgery
Hemangioma	3 (7.3%)	Left atrium (1), right atrium (1), right atrioventricular septal area (1)	Surgery (2)
			Biopsy (1)
Angioleiomyolipoma	1 (2.4%)	Right atrium	Surgery
Ventricular aneurysm	1 (2.4%)	Left ventricle	Surgery
Thrombus	1 (2.4%)	Left ventricle	Surgery
Unkown benign tumor	3 (7.3%)	Right atrium (1), left ventricle (2)	Follow-up (≥ 28 moths)
Tuberculous pericarditis	2 (4.9%)	Pericardium	biopsy
Nonspecific pericarditis	2 (4.9%)	Pericardium	Follow-up (Lesions disappeared after anti-inflammatory treatment)

**Figure 1:**
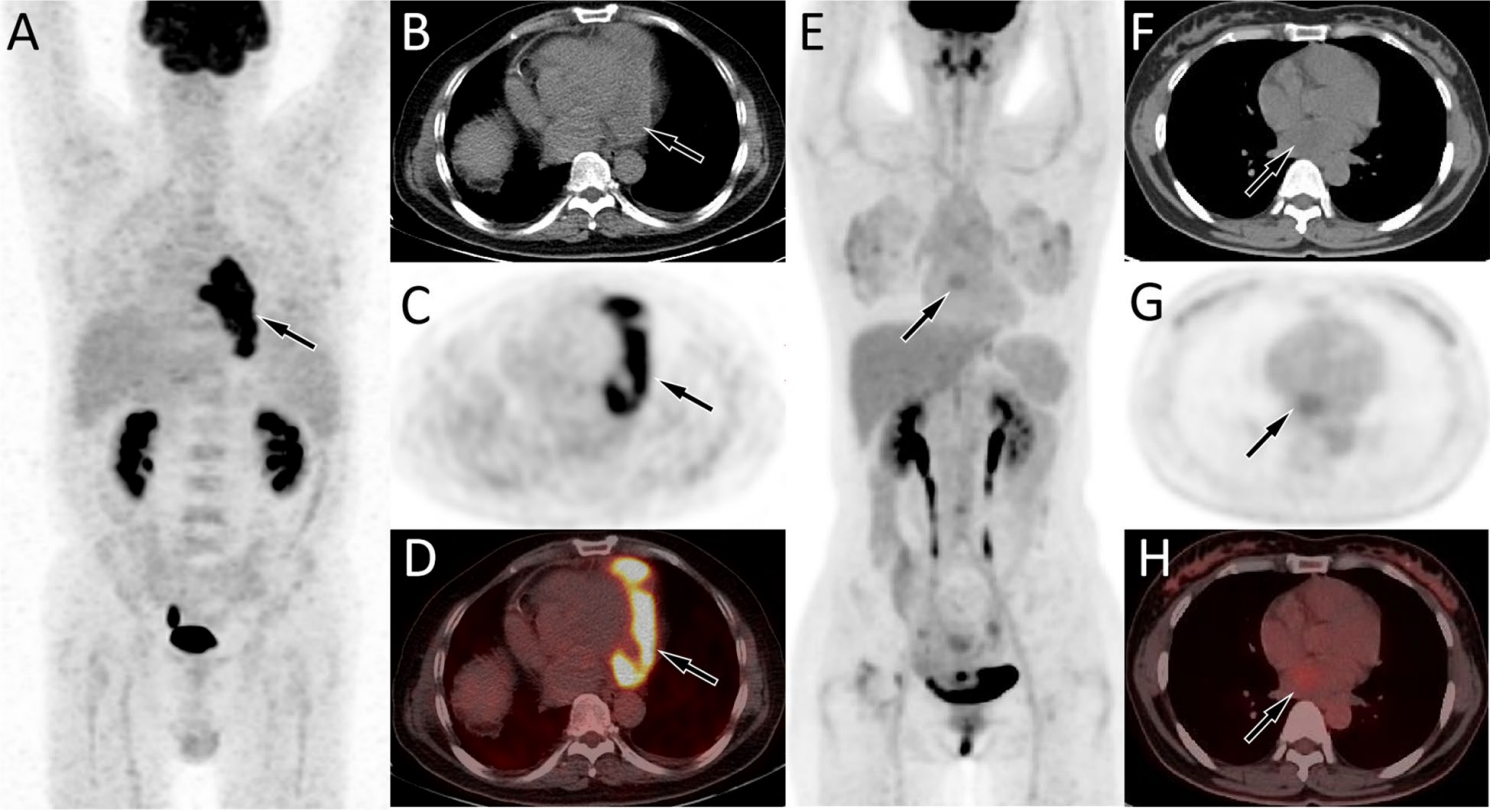
(**A**–**D**) A 50 year old male patient underwent CT examination due to breathing difficulties and found an isodensity soft tissue mass in the pericardium. To further evaluate the nature of the mass, PET/CT examination was performed, which was later confirmed by pathology as tuberculous pericarditis. The maximum intensity projection (MIP, **A**) showed a signifcantly increased ^18^F-FDG uptake in the heart region (arrow). Axial CT (**B**), PET (**C**) and PET/CT(**D**) showed that the lesion with high ^18^F-FDG uptake on the MIP map, which is isodense soft tissue mass located in the left part of the pericardium, with SUVmax, SUVmean, and MTV being 11.06, 7.12, and 25.43, respectively (arrows). (**E**–**H**) A 47-year-old woman with palpitation and dyspnea underwent echocardiography to find a right atrial mass. PET/CT was performed to further evaluate the nature of the tumor, which was pathologically confirmed as myxoma. The MIP map showed a lesion with slightly increased ^18^F-FDG uptake in the heart region (**E**, arrow). Axial CT (**F**), PET (**G**) and PET/CT (**H**) showed that the lesion was isodensity and located in the right atrium, with SUVmax, SUVmean, and MTV being 3.2, 1.7, and 6.48, respectively (arrows).

**Figure 2:**
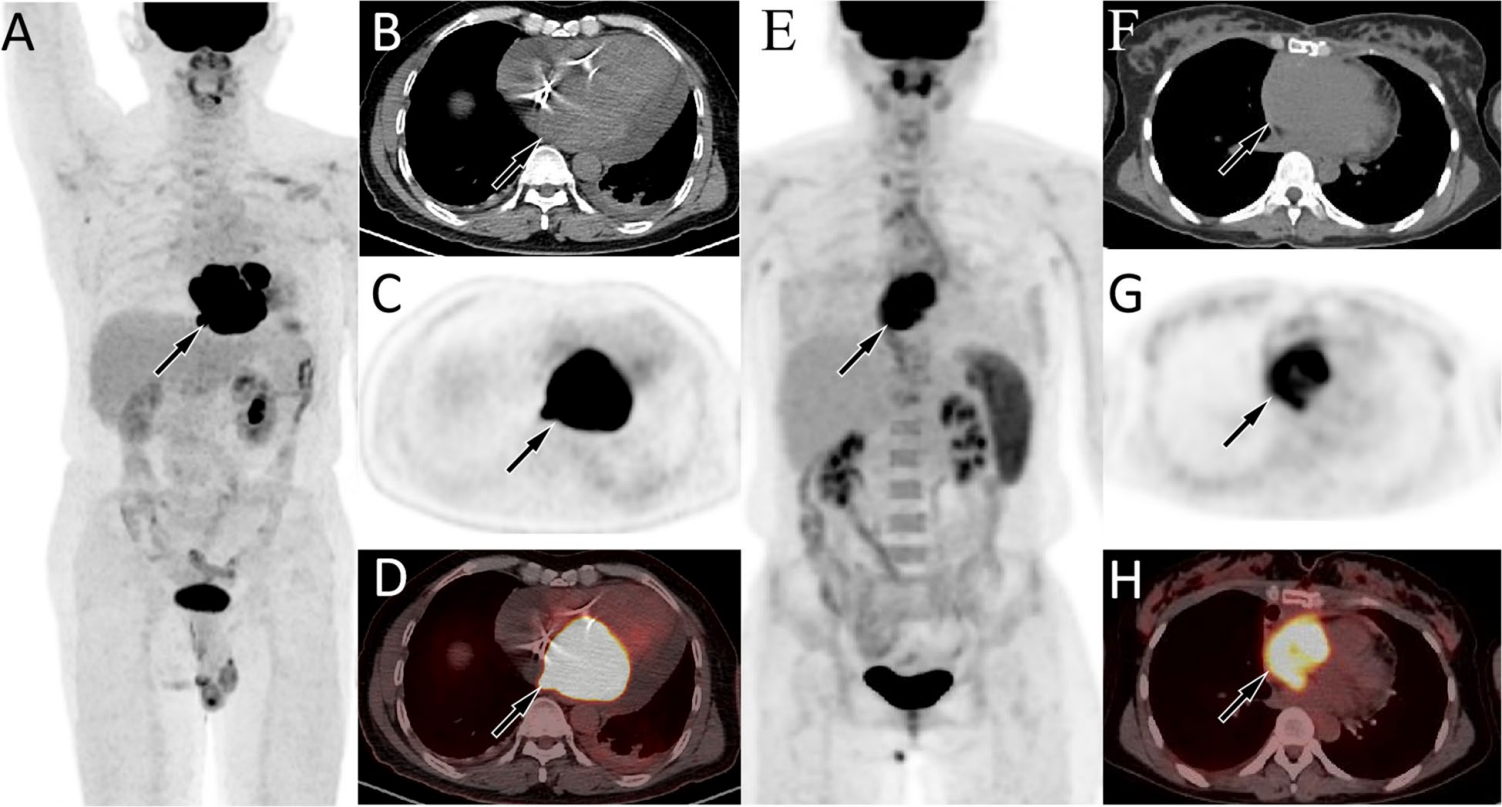
(**A**–**D**) A 67-year-old male patient with chest tightness and shortness of breath underwent echocardiography which revealed a left atrial mass, followed by PET/CT to assess its nature, and was subsequently confirmed as diffuse large B-cell lymphoma. The maximum intensity projection (MIP, **A**) showed a signifcantly increased ^18^F-FDG uptake in the heart region (arrow). Axial CT (**B**), PET (**C**) and PET/CT(**D**) showed that the lesion with high ^18^F-FDG uptake on the MIP map, which is isodense soft tissue mass located in the left atrium, with SUVmax, SUVmean, and MTV being 29.5, 15.49, and 67.52, respectively (arrows). (**E**–**H**) A 30-year-old woman with chest tightness and pain for 1 month underwent echocardiography and found a right atrial occupation, followed by PET/CT. The MIP map showed a lesion with significantly increased ^18^F-FDG uptake in the heart region (**E**, arrow). Axial CT (**F**), PET (**G**) and PET/CT (**H**) showed that the lesion was isodensity and located in the right atrium, with SUVmax, SUVmean, and MTV being 10.9, 6.4, and 46.29, respectively (arrows), which was later confirmed by pathology as angiosarcoma.

There was no significant difference in CT density between the benign group and the malignant group, both of which can present homogeneous and heterogeneous density. In the benign group, lesions that occur in the pericardium may exhibit mildly increased ^18^F-FDG uptake, while a few may also present obviously increased ^18^F-FDG uptake, such as tuberculous pericarditis (as shown in Fig. 1A–D). Benign tumors or tumor-like lesions originating from the atrium or ventricle often present mildly ^18^F-FDG uptake or no ^18^F-FDG uptake on PET/CT (Fig. 1E–H). As for the malignant group, tumors originating from pericardium, atria and ventricles mostly showed significantly increased ^18^F-FDG uptake (Fig. 2). Among the cases enrolled in our study, only one low-grade malignant cardiac tumor, hemangiopericytoma, showed mildly increased ^18^F-FDG uptake.

### Semi-quantitative parameter comparison and diagnostic performance of ^18^F-FDG PET/CT

**Figure 3:**
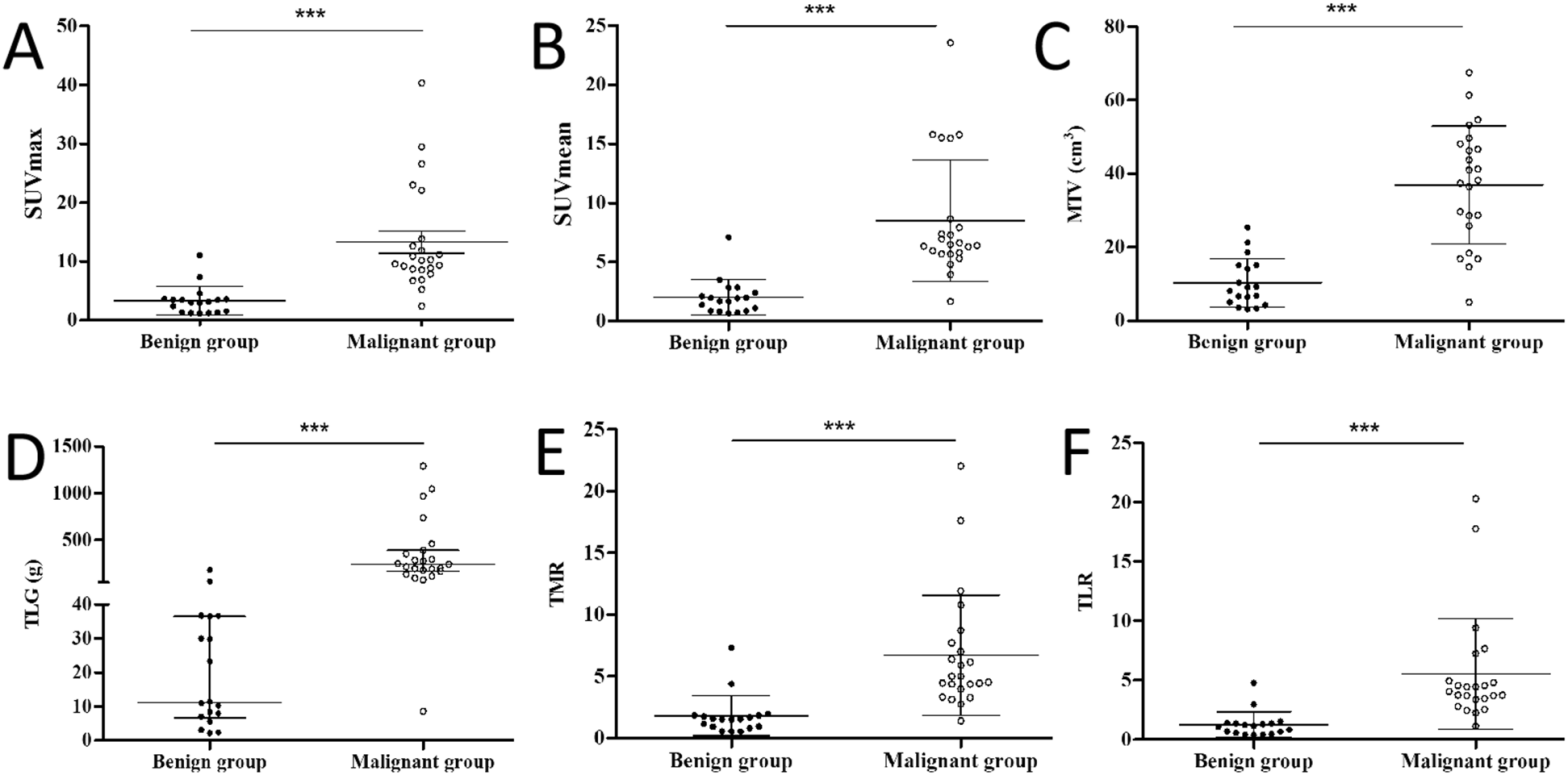
The SUVmax (**A**), SUVmean (**B**), TLG (**C**), MTV (**D**), TMR (**E**) and TLR (**F**) distribution in benign and malignant heart/pericardial lesions, and the malignant group were significantly higher than that of benign group (All *p*-values are less than 0.001). Notes: SUVmax, maximum standardized uptake value; SUVmean, mean standardized uptake value; MTV, metabolic tumor volume; TLG, total lesion glycolysis; TMR, the SUVmax of tumor-to-mediastinal background ratio; TLR, the SUVmax of tumor-to-liver background ratio; ***p < 0.001.

**Figure 4:**
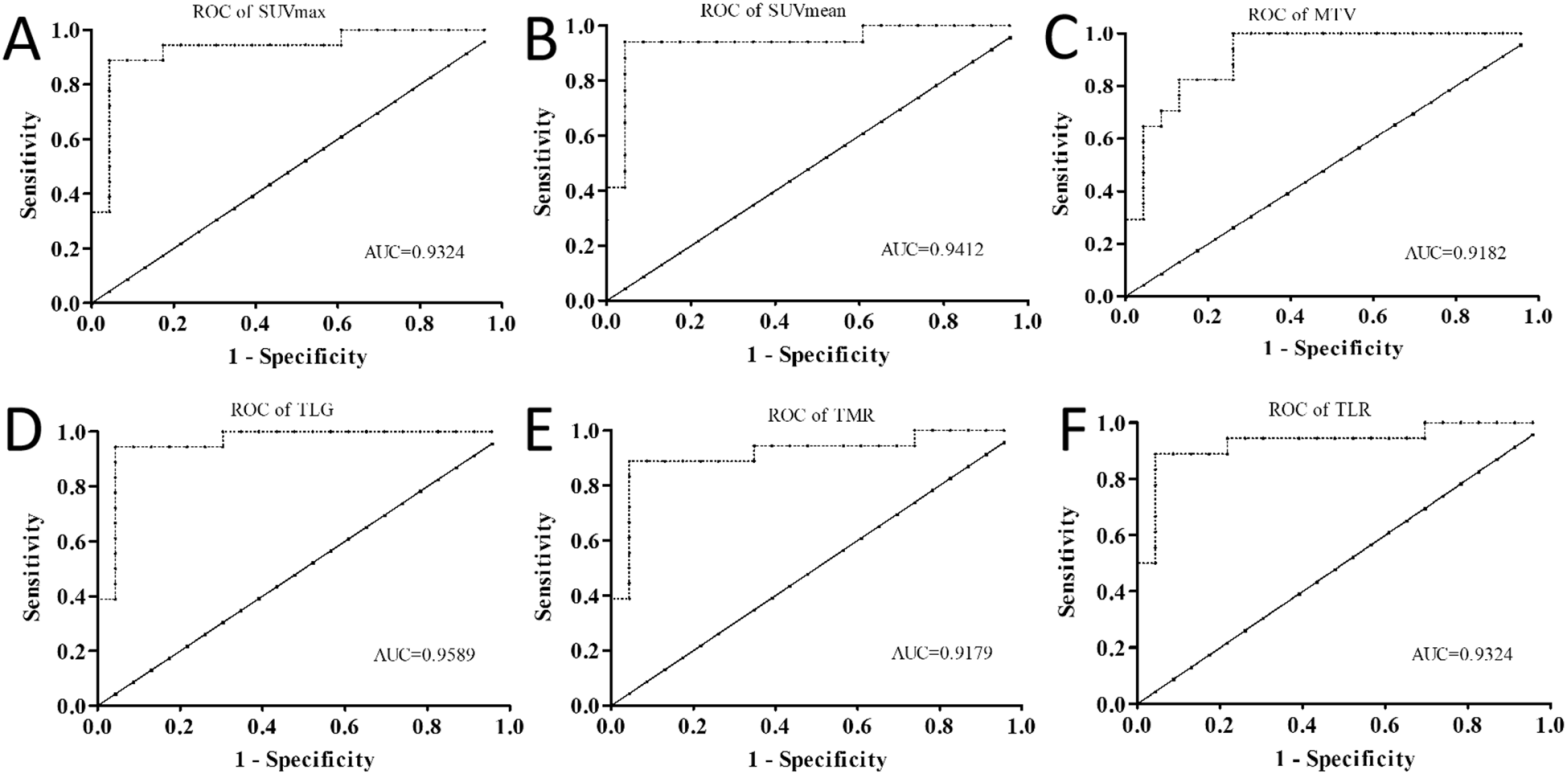
The ROCs of SUVmax (**A**), SUVmean (**B**), TLG (**C**), MTV (**D**), TMR (**E**) and TLR (**F**) in differentiating malignant tumor from benign lesions. Notes: SUVmax, maximum standardized uptake value; SUVmean, mean standardized uptake value; MTV, metabolic tumor volume; TLG, total lesion glycolysis; TMR, the maximum tumor-to-mediastinal background ratio; TLR, the maximum tumor-to-liver background ratio; ROC, receiver operating characteristic curve; AUC, area under the curve.

The mean/median SUVmax, SUVmean, MTV, TLG, TMR, and TLR for the benign and malignant groups were 3.35 versus 13.31, 2.03 versus 8.50, 10.37 versus 36.99, 11.23 versus 242.43, 1.81 versus 6.72 and 1.26 versus 5.54, respectively (Table 1). The detailed distribution of SUVmax, SUVmean, MTV, TLG, TMR and TLR values in benign and malignant heart or pericardial lesions is shown in Fig. 3. The ROC analysis showed that all PET/CT metabolic parameters were statistically different in differentiating benign and malignant cardiac or pericardial masses, and all *p*-values were less than 0.05. The AUC of SUVmax, SUVmean, MTV, TLG, TMR, and TLR were 93.24%, 94.12%, 91.82%, 95.89%, 91.79%, 93.24%, respectively (Fig. 4). Among these semi-quantitative parameters, SUVmean and TLG had the same and the best diagnostic performance in differentiating benign and malignant cardiac/pericardial lesions, with sensitivity, specificity and accuracy of 95.7%, 94.4% and 95.1%, respectively. The diagnostic performance of all semi-quantitative parameters in differentiating benign and malignant cardiac/pericardial lesions is detailed in Table 3.

**Table 3:** The diagnostic performance of PET/CT parameters in differentiating benign and malignant cardiac/pericardial masses (n = 41).

Parameters	Cut-off value	Sensitivity (%)	Specificity (%)	Accuracy (%)	AUC
SUVmax	4.93	91.3 (21/23)	94.4 (17/18)	92.7 (38/41)	93.2
SUVmean	3.73	95.7 (22/23)	94.4 (17/18)	95.1 (39/41)	94.1
MTV	16.05	91.3 (21/23)	94.4 (17/18)	92.7 (38/41)	93.5
TLG	69.45	95.7 (22/23)	94.4 (17/18)	95.1 (39/41)	95.6
TMR	2.37	87.0 (20/23)	94.4 (17/18)	90.2 (37/41)	91.8
TLR	1.90	91.3 (21/23)	94.4 (17/18)	92.7 (38/41)	93.2

## Discussion

The incidence of cardiac tumors is low, the clinical symptoms of patients are not typical, the results of laboratory tests lack specificity, and routine imaging tests have limitations in differentiating benign and malignant cardiac tumors. ^18^F-FDG PET/CT adds metabolic information of tumor cells on the basis of anatomic images, which can be used as a supplementary examination method when it is difficult to differentiate benign and malignant tumors by routine imaging. SUVmax is a core metabolic parameter of ^18^F-FDG PET/CT, which is used to measure the uptake of ^18^F-FDG by tumors or other abnormal lesions, which represents the maximum concentration of ^18^F-FDG uptake in the tumor area^16^. Since most malignant tumors, including breast cancer, ovarian cancer, lymphoma and other malignant tumors, have the characteristics of high metabolism, most of which show a significant increase in the uptake of ^18^F-FDG on PET, resulting in a higher SUVmax^17–19^. A previous study showed that when the cutoff value of SUVmax is 3.5, the diagnostic performance of differentiating benign and malignant cardiac or pericardial tumors is the best^9^. Our results showed that the SUVmax of cardiac malignant tumors was significantly higher than that of benign tumors, but different from the results of the previous study, our results showed that when the cut-off value of SUVmax was 4.93, the diagnostic efficiency was the best.

Different from SUVmax, SUVmean is the mean of all voxels in VOI, and its main advantage is that it is less affected by image noise, so that better detection repeatability can be obtained. Therefore, SUVmean is also widely used in the study of benign and malignant tumor identification and prognosis assessment^20,21^. MTV is used to quantify the metabolic activity of the tumor, which provides information on the overall biological activity of the tumor, considering not only the most active part of the tumor, but also the entire scope of the tumor, and can be used to evaluate the volume and biological characteristics, diagnosis, staging, prognosis assessment, and treatment response monitoring of the tumor^22,23^. TLG is the product of MTV and SUVmean, which represents the total uptake of ^18^F-FDG in the whole tumor. Combined with the volume and average metabolic activity of the tumor, TLG provides information about the systemic metabolic load of the tumor, which can be used to evaluate the biological activity and prognosis of the tumor, and guide the selection of treatment strategies^24^. Therefore, besides SUVmax, our current study further evaluated the diagnostic performance of various PET metabolic parameters including SUVmean, MTV and TLG in differentiating benign and malignant heart/pericardial lesions. The results showed that that SUVmean, TLG and MTV of malignant tumors of the heart/pericardium were significantly higher than those of benign lesions, and all of these metabolic parameters showed high diagnostic performance.

Moreover, since the background of mediastinum and liver is related to tumor uptake, and the benign and malignant tumors can be evaluated by calculating the SUVmax ratio of tumor to mediastinum (TBR) and the SUVmax ratio of tumor to liver (TLR)^13,14^. Compared with other metabolic parameters such as SUVmax in PET/CT, TBR and TLR are less affected by body composition and image reconstruction methods, and therefore are also used as parameters in the differentiation of benign and malignant tumors^25,26^. The current research results show that malignant tumors of the pericardium or heart have larger TMR and TLR values than benign lesions, and have high sensitivity and specificity in differentiating between benign and malignant lesions, which is equivalent to the diagnostic performance of SUVmax.

SUVmax, as the most commonly used PET metabolic parameter, is not affected by the delineation threshold, and is an important parameter for differentiating benign and malignant cardiac tumors. However, SUVmax alone could not completely evaluate tumor volume and metabolic status. Compared with previous studies^8,9,13,14^, we used multiple parameters of PET/CT, including SUVmax, SUVmean, MTV, TLG, TMR, and TLR, to semi-quantitatively evaluate the benign and malignant nature of pericardial or cardiac lesions. All of these parameters showed high accuracy in differentiating benign and malignant lesions of the heart or pericardium, among which, SUVmean and MTV had the highest diagnostic performance in differentiating cardiac/pericardial benign from malignant lesions, suggesting that SUVmax may not be the best reference for evaluating the nature of cardiac or pericardial tumors.

A limitation of this study is the small sample size due to the relative rarity of cardiac tumors. Second, because cardiac muscle would appear to be physiological uptake, but based on the retrospective nature of our study, no special patient preparation, such as a high fat/low carbohydrate diet or heparin intervention, was performed for detecting patients with cardiac tumors.

## Conclusion

^18^F-FDG PET/CT metabolic parameters including SUVmax, SUVmean, TLG, MTV, TMR and TLR can be used to semi-quantitatively evaluate the benign and malignant of cardiac or pericardial tumors, among which SUVmean and MTV have the highest diagnostic accuracy.

## Data Availability

The datasets used and materials during the current study are available from the corresponding author on reasonable request.
